# The Future of the RTS,S/AS01 Malaria Vaccine: An Alternative Development Plan

**DOI:** 10.1371/journal.pmed.1001994

**Published:** 2016-04-12

**Authors:** Roly Gosling, Lorenz von Seidlein

**Affiliations:** 1 Global Health Group, University of California, San Francisco, San Francisco, California, United States of America; 2 Mahidol-Oxford Tropical Medicine Research Unit (MORU), Bangkok, Thailand

## Abstract

Roly Gosling and Lorenz von Seidlein consider a potential future development plan for the RTS,S/AS01 malaria vaccine.

**Summary Points**

- RTS,S/AS01 is the falciparum malaria vaccine candidate that is most advanced in development, globally.
- In a large clinical trial in sub-Saharan African children, the protection conferred by RTS,S/AS01 was found to rapidly decline, particularly in infants.
- Although RTS,S/AS01 was approved by the European Medicines Agency for active immunization of children aged 6 weeks to 17 months against malaria, the WHO did not recommend the inclusion of RTS,S/AS01 in the Expanded Programme of Immunisations (EPI).
- Instead of aiming for inclusion of the vaccine in EPI, we propose that the future development of RTS,S/AS01 could take advantage of its high transient protective efficacy.
- Adding the vaccine to intensive malaria elimination strategies in low-endemicity areas could be the critical factor in interrupting transmission.

The malaria vaccine candidate, RTS,S/AS01 (also known as Mosquirix), consists of hepatitis B surface antigen virus-like particles, incorporating a portion of the *Plasmodium falciparum*-derived circumsporozoite protein and a liposome-based adjuvant. The clinical development of the malaria vaccine RTS,S/AS01 reached a critical stage in 2015 with the publication of the results of a large, pivotal trial that started in 2009 and enrolled over 15,000 infants and young children [1]. The findings of the trial were sobering (see Box 1). In both infants aged 6 to 12 weeks and young children 5 to 15 months old, vaccine efficacy waned rapidly. The addition of a booster dose 20 months after the first dose increased protection only slightly. Overall, the vaccine was considered safe, but significantly more vaccine recipients in the 5–17-month-old age group experienced meningitis compared to children who received the control vaccines. The detection of meningitis as a possible safety signal requires follow-up during post-registration studies.

**Box 1. Key Information about RTS,S/AS01**

1. In a large Phase III trial of RTS,S/AS01 at 11 sites in seven sub-Saharan African countries, 8,922 children and 6,537 young infants were included in the modified intention-to-treat analyses.
  ○ Vaccine efficacy (VE) against clinical malaria in infants between the ages of 6 and 12 weeks, following three primary doses, declined from 27.0% (95% confidence interval [CI] 21.1 to 32.5) to 20.3% (13.6 to 26.5) to 18.3% (11.7 to 24.4) during 20, 32, and 48 months of follow-up, respectively [1]. The addition of a booster dose increased the VE from 18.3% to 25.9%, (19.9 to 31.5). The vaccine provided no significant protection against severe malaria.
  ○ Evidence for protection in young children 5–17 months of age was more encouraging. The VE against clinical malaria in children aged 5–17 months following three primary doses was 45.1% (41.4 to 48.7), 35.2% (30.5 to 39.5), and 28.3% (23.3 to 32.9) during 20, 32, and 48 months of follow-up, respectively [1]. The addition of a booster dose at 18 months increased the VE from 28.3% to 36.3% (31.8 to 40.5). The vaccine provided no significant protection against severe malaria, perhaps related to its low incidence in trial participants. Protection waned over time, with the highest efficacy noted soon after vaccination.
  ○ Similar numbers of severe adverse events were detected between the vaccine and control groups, with the exception of meningitis [1]. In the children aged 5–17 months, there were 21 cases of meningitis in the 5,948 RTS,S/AS01 recipients and one in the 2,974 controls. This signal is cause for concern, remains unexplained, and requires careful follow-up should RTS,S/AS01 be rolled out. No imbalance in cases of meningitis was noted in the infants.
2. The safety and efficacy of RTS,S/AS01 against malaria has also been demonstrated in small numbers of North American adults [2] and African adults [3]. Safety and immunogenicity has been shown in de-escalating age groups in sub-Saharan Africa [4–7]. The protective efficacy of RTS,S/AS01 has not been evaluated in Asian or South American populations.
3. Each vaccine dose is expected to cost around US$5 [8]. A three-dose regimen with a booster will cost about US$20, not including the costs of delivery. Note that US$20 is more than half the average annual per capita health expenditure in low-income countries, which was US$37 in 2013 [9].

In July 2015, RTS,S/AS01 was approved by the European Medicines Agency for immunization of children aged 6 weeks to 17 months against malaria under Article 58,12 [10]. This assessment is aimed at supporting regulatory agencies in endemic countries for licensure of the vaccine. But without strong endorsement from the WHO, it is doubtful that donors like GAVI or UNICEF will purchase the vaccine and unlikely that malaria control programmes will allocate resources to integrate the vaccine in their relevant programmes. In their October 2015 meeting, the WHO's Strategic Advisory Group of Experts on Immunization and the Malaria Policy Advisory Committee jointly decided not to recommend the use of RTS,S/AS01 in infants, i.e., within the Expanded Programme of Immunisations (EPI) [11]. To recommend the use of the vaccine in the older age group (5 to 17 months) in malaria-endemic countries, the committees will require more data on the feasibility, safety, and effectiveness of the vaccine. Instead, the assembled experts recommended pilot implementations that “use the four-dose schedule of the malaria vaccine in three to five distinct epidemiological settings” [11]. It will be critical to follow the participants over an extended period to monitor long-term safety and vaccine efficacy (VE) in the individual and amongst sequential cohorts, in case the vaccine selects out certain genetic strains of *P*. *falciparum*, leaving vaccine-resistant strains remaining.

Before the limited funds available for public health interventions are invested into the roll-out of RTS,S/AS01 vaccine programmes, it has to be convincingly shown that in a real-life setting, over a 10 to 15 year period, a cohort of children vaccinated early in life develop significantly less malaria-related morbidity and mortality, compared to an unvaccinated cohort. Even a modest programme that follows participants for only 5 years would cost well above the hundred-million-dollar mark. In the absence of donors, enthusiasm for the vaccine in the malaria community will likely wane, and there is a real possibility that the development of RTS,S/AS01 will stall and we will have to wait a decade or more for the next generation of malaria vaccines.

Over the last three decades, probably well above half a billion dollars has been spent in the development of RTS,S/AS01. When RTS,S/AS01 was still in its early phase, falciparum malaria was hyper-endemic in much of sub-Saharan Africa. The highest burden of the disease was in very young children, with virtually every resident in endemic areas infected at some stage during early life. The large majority of malaria cases occurred in children under 5 years, and children who survived to school age had substantial protective immunity. Twenty years ago, it was imperative to develop a vaccine that would protect children as early as possible; ideally such a vaccine would be included in the EPI. But since the beginning of this century, there have been profound changes in malaria epidemiology. In many endemic regions, malaria transmission has been reduced to historically low levels, coinciding with unprecedented funding of malaria control programmes that has resulted in the roll-out of long-lasting insecticide-treated bednets and the increasing availability of appropriate case management where it is needed [12]. With reduced exposure to malaria, children are older before they have their first malaria episode, or they may not become infected at all. Consequently, the age group with the greatest malaria burden has shifted from under five-year-olds to school-age children and beyond. If the RTS,S/AS01 vaccine could provide early and lifelong immunity, this shift in malaria epidemiology would be a moot point. Unfortunately this is not the case. RTS,S/AS01 vaccination provides little protection in infants; the better efficacy noted in older children wanes rapidly.

Once the malaria epidemiologic transition in sub-Saharan Africa became obvious [13,14], malaria control strategies including the proposed programmatic use of RTS,S/AS01 should have been re-examined. Alternative uses of the vaccine outside the EPI in sub-Saharan Africa should have been explored, but this did not happen. There is no well-established, cost-effective method of vaccinating older age groups outside the EPI schedule. Keeping RTS,S/AS01 in the EPI track allows a producer to forecast with a high level of accuracy the demand for the vaccine for years to come, generating a steady income stream. In contrast, planning to vaccinate school-age children and other age groups for purposes such as outbreak control or malaria elimination may have been considered an unacceptable level of uncertainty.

The termination of the RTS,S/AS01 development would be a loss for malaria elimination efforts. With all the attention given to the disappointing waning of vaccine-induced immunity, the potential benefit of the high initial protection afforded by the vaccine has been all but forgotten. Neafsey et al. recently estimated the protective efficacy of RTS,S/AS01 in relation to the parasite genotype at the circumsporozoite locus. The vaccine affords the highest protection against parasites with a genotype that matches the circumsporozoite protein allele on which the vaccine is based [15]. Against matched parasite strains, which make up only 10% of the total parasite population in the trial, the estimated protective efficacy during the first 50 days after vaccination reached close to 100% and stayed over 75% for the first 100 days following vaccination. The protective efficacy of RTS,S/AS01 against parasite strains that are not a perfect match peaked around 75% and stayed above 50% for the first 200 days after vaccination [15]. This is a major achievement, and while it does not suffice to justify including RTS,S/AS01 in the EPI, it may provide a critical breakthrough where short-term suppression of malaria transmission is needed.

In many Asian countries and, specifically, in the Greater Mekong Subregion (GMS) where artemisinin resistance is rapidly spreading [16], there exists a strong will and funding to rapidly eliminate falciparum malaria [17]. If multidrug-resistant *P*. *falciparum* strains are transported through human migration and introduced into current and former high transmission areas in sub-Saharan Africa, a surge in malaria-related illness and death, as was seen during the spread of chloroquine resistance in the late 1900s, might be seen. Currently strategies employed to eliminate *P*. *falciparum* in the GMS include targeted malaria elimination (TME) that delivers appropriate case management by village health workers, vector control, and targeted mass drug administration. TME is a time-consuming process requiring several months of resource-intensive implementation to reach high coverage, and the elimination of parasitaemia using anti-malarial drugs is only short term. There is a possibility that with travel and migration, persons yet to be treated re-introduce infections into treated villages, leading to a gradual resurgence. Extending the parasitaemia-free period in the majority of villagers for as short a period as 200 days could increase the chances of achieving the interruption of malaria transmission. Addition of mass RTS,S/AS01 vaccinations to the TME arsenal could provide this much-needed additional protection.

Reactive ring vaccinations are another alternative strategy that could make use of the short-term protection gained from RTS,S/AS01. When time is critical and/or the availability of a vaccine is limited, it is most efficient to vaccinate the population at highest risk of becoming infected. People living around a malaria patient are at an increased risk to become infected and therefore have a higher need for protection, compared to people living in areas where no cases have been detected [18]. In the reactive ring vaccination strategy, a team would visit the patient and vaccinate household members and neighbours. Ring vaccinations target all people within a radius around a geographically defined population. This strategy has played a critical role in the eradication of smallpox [19,20] and was used with great success in the recent evaluation of an Ebola vaccine candidate [21]. If re-introduction of malaria could be prevented for even a limited period, at least in theory, transmission could be interrupted permanently. Similarly, it has been recognised that certain occupational groups, e.g., forest workers, are at an increased risk of becoming infected with malaria. Vaccinating high-risk occupational groups could result in protection for their benefit and reduce the transmission risk to people living in their neighbourhood.

Before such alternative pilot programs can take place, additional research is needed (see also Box 2). The large RTS,S/AS01 trial in sub-Saharan Africa vaccinated children; the dose required to vaccinate adults is less well established. It is essential to establish the safety and immunogenicity of the intended vaccine dose in populations other than African children [1] and adults [3] through bridging studies. Such an undertaking has been made much easier by the recent analysis of the immune responses to the circumsporozoite protein, which found a close correlation between anti-circumsporozoite antibody titers and vaccine-induced protection against malaria [22]. An antibody titre of 121 EU/ml (95% CI 98 to 153) was associated with prevention of 50% of malaria infections. These findings will help answer the question of what vaccine dose in different populations is required to trigger a protective immune response against malaria. There is also the remote possibility that the concomitant administration of RTS,S/AS01 with antimalarial drugs could lead to a reduced immune response. Concomitant use of chloroquine may reduce the antibody response to intradermal rabies vaccine administered for pre-exposure vaccination [23]. Such interference is unlikely, considering that RTS,S/AS01 is a subunit vaccine, yet needs to be ruled out prior to large scale use of RTS,S/AS01 in conjunction with antimalarial treatment.

**Box 2. Prerequisites for Use of RTS,S/AS01 in Asian and Pacific Regions**

- Dose selection for adults
- Safety and immunogenicity data for populations other than sub-Saharan African children and adults
- Confirmation that the co-administration of antimalarial drugs (e.g., piperaquine) does not reduce vaccine-induced immunogenicity
- Efficacy against American, Asian, and Pacific strains of *P*. *falciparum*

Finally, there remains a need to assess whether RTS,S/AS01-triggered immunity provides similar protection against the parasites with potentially differently shaped circumsporozoite proteins than the ones circulating in sub-Saharan Africa. This question may be best addressed in large-scale effectiveness studies in the Greater Mekong Subregion.

In summary, a new tool for malaria control, RTS,S/AS01, is now available. Although its performance is somewhat disappointing in sub-Saharan African children, the vaccine’s short-term efficacy could potentially be used in other regions and other age groups. Global efforts are currently underway to eliminate malaria, with a special focus in Southeast Asian areas with low malaria incidence and high antimalarial drug resistance. Integration of RTS,S/AS01 into elimination strategies may improve the chances of success.

## Abbreviations

EPI: Expanded Programme of Immunisations
GMS: Greater Mekong Subregion
TME: targeted malaria elimination
VE: vaccine efficacy
